# Sustained aviremia despite anti-retroviral therapy non-adherence in male children after in utero HIV transmission

**DOI:** 10.1038/s41591-024-03105-4

**Published:** 2024-06-06

**Authors:** Nomonde Bengu, Gabriela Cromhout, Emily Adland, Katya Govender, Nicholas Herbert, Nicholas Lim, Rowena Fillis, Kenneth Sprenger, Vinicius Vieira, Samantha Kannie, Jeroen van Lobenstein, Kogielambal Chinniah, Constant Kapongo, Roopesh Bhoola, Malini Krishna, Noxolo Mchunu, Giuseppe Rubens Pascucci, Nicola Cotugno, Paolo Palma, Alfredo Tagarro, Pablo Rojo, Julia Roider, Maria C. Garcia-Guerrero, Christina Ochsenbauer, Andreas Groll, Kavidha Reddy, Carlo Giaquinto, Paolo Rossi, Seohyun Hong, Krista Dong, M. Azim Ansari, Maria C. Puertas, Thumbi Ndung’u, Edmund Capparelli, Mathias Lichterfeld, Javier Martinez-Picado, John C. Kappes, Moherndran Archary, Philip Goulder

**Affiliations:** 1 Queen Nandi Regional Hospital, Empangeni, South Africa; 2 HIV Pathogenesis Programme, Doris Duke Medical Research Institute, Nelson R. Mandela School of Medicine, University of KwaZulu-Natal, Durban, South Africa; 3 Department of Paediatrics, University of KwaZulu-Natal, Durban, South Africa; 4 Department of Paediatrics, University of Oxford, Oxford, UK; 5 Africa Health Research Institute, Durban, South Africa; 6 Harry Gwala Regional Hospital, Pietermaritzburg, South Africa; 7 General Justice Gizenga Mpanza Regional Hospital, Stanger, South Africa; 8 Mahatma Gandhi Memorial Hospital, Durban, South Africa; 9 Clinical Immunology and Vaccinology Unit, IRCCS, Ospedale Pediatrico Bambino Gesù, Rome, Italy; 10 Probiomics S.r.l., Rome, Italy; 11 University of Rome Tor Vergata, Rome, Italy; 12 Fundación de Investigación Biomédica Hospital 12 de Octubre, Instituto de Investigación 12 de Octubre (imas12), Madrid, Spain; 13 Department of Pediatrics, Infanta Sofia University Hospital and Henares University Hospital Foundation for Biomedical Research and Innovation, Madrid, Spain; 14 Universidad Europea de Madrid, Madrid, Spain; 15 LMU University Hospital, Munich, Germany; 16 IrsiCaixa AIDS Research Institute, Barcelona, Spain; 17 University of Alabama at Birmingham, Birmingham, AL USA; 18 TU Dortmund University, Dortmund, Germany; 19 University of Padova, Padua, Italy; 20 Ragon Institute of MGH, MIT and Harvard, Boston, MA USA; 21 Nuffield Department of Medicine, University of Oxford, Oxford, UK; 22 Consorcio Centro de Investigación Biomédica en Red de Enfermedades Infecciosas (CIBERINFEC), Instituto de Salud Carlos III, Madrid, Spain; 23 Division of Infection and Immunity, University College London, London, UK; 24 University of California San Diego, San Diego, CA USA; 25 Catalan Institution for Research and Advanced Studies (ICREA), Barcelona, Spain; 26 Infectious Diseases and Immunity Department, University of Vic-Central University of Catalonia, Vic, Spain; 27 Birmingham Veterans Affairs Medical Center, Research Service, Birmingham, AL USA

**Keywords:** Predictive markers, HIV infections, HIV infections, Outcomes research

## Abstract

After sporadic reports of post-treatment control of HIV in children who initiated combination anti-retroviral therapy (cART) early, we prospectively studied 284 very-early-cART-treated children from KwaZulu-Natal, South Africa, after vertical HIV transmission to assess control of viremia. Eighty-four percent of the children achieved aviremia on cART, but aviremia persisting to 36 or more months was observed in only 32%. We observed that male infants have lower baseline plasma viral loads (*P* = 0.01). Unexpectedly, a subset (*n* = 5) of males maintained aviremia despite unscheduled complete discontinuation of cART lasting 3–10 months (*n* = 4) or intermittent cART adherence during 17-month loss to follow-up (*n* = 1). We further observed, in vertically transmitted viruses, a negative correlation between type I interferon (IFN-I) resistance and viral replication capacity (VRC) (*P* < 0.0001) that was markedly stronger for males than for females (*r* = −0.51 versus *r* = −0.07 for IFN-α). Although viruses transmitted to male fetuses were more IFN-I sensitive and of higher VRC than those transmitted to females in the full cohort (*P* < 0.0001 and *P* = 0.0003, respectively), the viruses transmitted to the five males maintaining cART-free aviremia had significantly lower replication capacity (*P* < 0.0001). These data suggest that viremic control can occur in some infants with in utero–acquired HIV infection after early cART initiation and may be associated with innate immune sex differences.

## Abstract (web summary)

After early initiation of anti-retroviral therapy in infants infected with HIV-1 in utero, some children sustain undetectable virus levels, despite treatment interruption, which associated with distinct features of the transmitted virus.

## Main

Studies of adult post-treatment controllers, such as the VISCONTI^1^ or CHAMP cohorts^2–4^, suggest that early combination anti-retroviral therapy (cART) initiation, low levels of immune activation and effective natural killer (NK) cell-mediated immunity may be key features contributing to HIV cure or remission. By contrast, immune control of cART-naive adult HIV infection is associated with very rapid and high levels of immune activation after infection and an aggressive anti-viral immune response driven by HIV-specific CD8^+^ T cell activity^5–7^. Although, from historical studies, virus-specific CD8^+^ T cells in cART-naive children are relatively ineffective in the first 1–2 years of life^8,9^, and untreated HIV disease progression is substantially more rapid in children than in adults^9,10^, it is proposed that early-cART-treated children may have a higher potential to achieve cART-free remission than adults^7^. First, cART can be initiated very early in the course of pediatric infection. Second, the highly regulated, tolerogenic early-life immune response^11^ reduces the numbers of activated CD4^+^ T cells available for HIV infection. Third, in early life, and in contrast with adults, NK responses are more effective mediators of control of HIV infection than virus-specific CD8^+^ T cells^12^. Finally, again contrasting with adult-to-adult transmission, the vertically transmitted virus typically has relatively low replication capacity^13^. This is associated with low levels of immune activation, low pro-viral DNA loads and slow progression in the recipient^14^. These factors, therefore, would tend to lower viral reservoir size and increase cure potential among children. Furthermore, the ability to study both mother and child at birth provides access to the transmitted ‘founder’ virus, a precious resource in understanding mechanisms of cART-free remission in those children who subsequently achieve it.

To identify which children, after early cART initiation, achieve undetectable HIV DNA loads and might, via additional immunotherapeutic interventions, ultimately achieve post-treatment control, we undertook an observational study between 2015 and 2023 of 284 mother–child pairs living with HIV (LWH) in KwaZulu-Natal, South Africa.

## Results

### Low baseline plasma HIV RNA loads in male infants

**Fig. 1:**
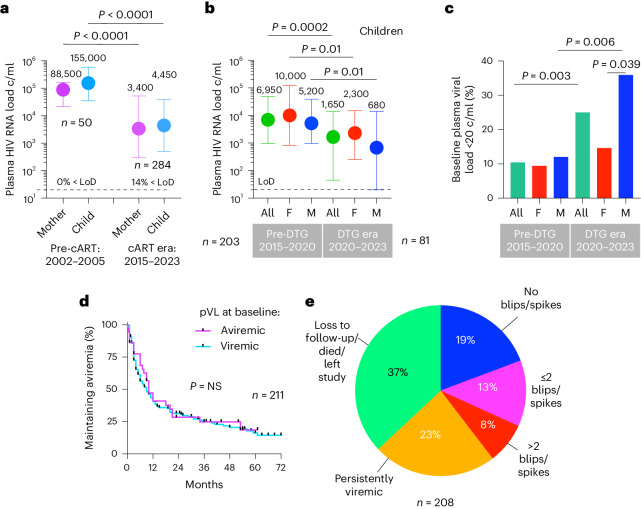
Impact of cART and changing cART regimens on baseline pVLs and maintenance of aviremia in pediatric HIV infection. **a**, pVLs at baseline in transmitting mothers and in utero–infected infants in the pre-cART era in KwaZulu-Natal, South Africa, 2002–2005, and in the cART era in KwaZulu-Natal, South Africa, 2015–2023. LoD, limit of detection: 20 HIV RNA copies per milliliter of plasma. Data are presented as median and IQR. **b**, Impact of DTG-based regimens on baseline pVLs in female and male in utero–infected infants. Data are presented as median and IQR. **c**, Impact of DTG-based regimens on the proportion of in utero–diagnosed infants with undetectable pVLs (<20 copies per milliliter). **d**, Time to viral rebound in study infants after suppression on cART: median 9 months in children aviremic at baseline (pVL <20 copies per milliliter) and 10 months in children viremic at baseline (pVL >20 copies per milliliter). The *P* value was determined using the log-rank Mantel–Cox test. **e**, Proportion of study cohort at 36 months after enrollment maintaining aviremia with or without viral blips/spikes; remaining on study but persistently viremic; or no longer on study as a result of loss to follow-up, death or relocation. The *P* values shown were determined using the two-sided Mann–Whitney *U*-test (**a**–**c**) and the log-rank Mantel–Cox test (**e**). c/ml, copies per milliliter; F, female; M, male.

Diagnosis of in utero infection was made via detection of HIV nucleic acid from two or more separately drawn blood samples. Anti-retroviral therapy (ART) was started at birth in all children (AZT/NVP in 68% of cases, NVP alone in 32%), and cART (AZT/3TC/NVP) was initiated after confirmation of infection (median 1 day and 11 days of age, respectively, in children diagnosed via point-of-care (POC) (*n* = 84) and standard-of care (SOC) (*n* = 200) testing). However, in more than 90% of cases (262/284), cART was effectively initiated in children before birth via placental transfer of medications taken by antenatal mothers^15,16^. We, therefore, observed low baseline/birth plasma HIV RNA loads (pVLs) in children, especially since April 2020, when dolutegravir (DTG)-based cART regimens became first line in pregnancy (Fig. 1a–c). In males, baseline pVLs were 0.7 log_10_ lower than in females, after adjusting for cART regimen and age at enrolment (*P* = 0.01; Extended Data Table 1), consistent with previous studies showing lower pVL in males in the first 2 years of life^17,18^, and 0.5 log_10_ lower total HIV DNA loads in male infants in this cohort, after adjusting for 25 other covariates^19^. However, maintenance of aviremia in children is generally short lived (Fig. 1d,e) because of challenges with cART adherence^20^. Thus, factors in addition to early cART initiation alone are required to achieve cART-free remission.

### Five atypical cases maintaining cART-free aviremia

**Fig. 2:**
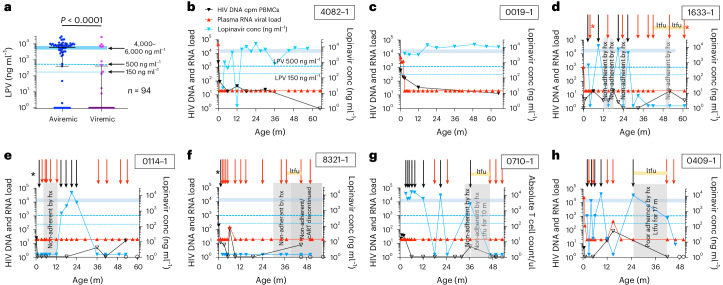
Maintenance of aviremia is dependent on cART adherence in most children and is independent of cART adherence in others. **a**, Cross-sectional analysis of 94 infants (median age 12 months) correlating pVLs with plasma concentrations of LPV. The normal range of LPV trough levels is 4,000–6,000 ng ml^−1^; <150 ng ml^−1^ is below the level of quantification. The *P* value shown was determined using the two-sided Mann–Whitney *U*-test. **b**, Example of a child who was cART non-adherent temporarily at 12 months but maintained aviremia without viral rebound. **c**, Example of a child who maintained aviremia without viral rebound and without any evidence of cART non-adherence. **d**–**h**, Five ‘atypical’ children, 60-1633-1 (**d**), 80-0114-1 (**e**), 60-8321-1 (**f**), 70-0710-1 (**g**) and 80-0409-1 (**h**), maintaining aviremia and mostly undetectable or not significantly different from undetectable total DNA loads despite persistent cART non-adherence by history and by analysis of plasma cART levels. Periods of loss to follow-up are shown as ‘ltfu’. Black arrows indicate timepoints at which LPV concentration was >500 ng ml^−1^; red arrows indicate timepoints at which LPV concentration was <500 ng ml^−1^; blue triangles indicate LPV concentration; red triangles indicate pVL copies per milliliter; solid black triangles indicate total HIV DNA load (cpm PBMCs) detected; open blank triangles indicate total HIV DNA load undetectable or not significantly different from undetectable; open circles indicate total HIV nucleic acid undetectable by GeneXpert; black asterisk indicates aviremia in association with prescribed cART that excluded LPV (that is, for 80-0114-1, AZT/3TC/NVP at 1 month of age); and red asterisks indicate cART non-adherent for the medications prescribed, but un-prescribed anti-retroviral drugs were detected at therapeutic levels at these timepoints, which could explain aviremia (see text for further specifics). conc, concentration; count/µl, count per microliter; m, months.

**Extended Data Fig. 1:**
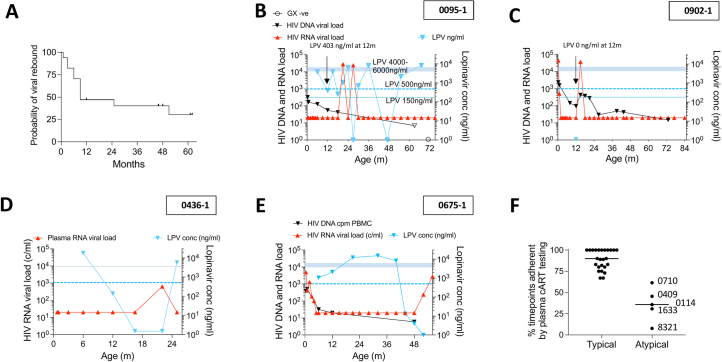
cART non-adherence in children identified by plasma cART testing. **a**. Time to viral rebound in children aviraemic but with low LPV levels at 12m (in Fig. 2a). **b-c**. Two examples of viral rebound following low levels of LPV detected at 12m (in Fig. 2a). **d-e**. Two examples of children followed longitudinally illustrating viral rebound following detection of low cART levels at later timepoints in the plasma. **f**. Proportion of timepoints when lopinavir levels were low in 25 ‘typical’ children maintaining aviraemia to age >48m without blips or spikes compared with the 5 ‘atypical’ children.

To better understand the relationship between pVL and cART adherence in the pediatric cohort, we analyzed plasma cART levels via liquid chromatography–tandem mass spectrometry (LC–MS/MS)^21^, in relation to contemporaneous pVL. We determined the levels of all 13 ART drugs prescribed in South Africa but focused on lopinavir (LPV) because all children were treated with LPV/ritonavir (RTV) from 1 month of age, and its plasma level is directly related to anti-viral activity. Even allowing for natural variation in drug absorption, LPV levels lower than 500 ng ml^−1^ are considered strong evidence of non-adherence during the 24 h immediately before blood sampling (12-h trough levels^22,23^ are typically 4,000–6,000 ng ml^−1^). In a cross-sectional analysis of 94 individuals at 12 months of age, as expected, LPV levels higher than 500 ng ml^−1^ were strongly associated with aviremia (*P* < 0.0001; Fig. 2a). However, 26% (*n* = 17) of 65 aviremic individuals had low LPV levels. Analysis of subsequent timepoints (Extended Data Fig. 1) showed, in most of these children, either viral rebound within 12 months or sustained aviremia after cART resumption. The 25 children who were persistently aviremic to age ≥48 months, excluding the five atypical individuals discussed further below, were cART adherent at 90% of timepoints (Fig. 2b,c, Extended Data Fig. 1f and Extended Data Table 2).

Five children, however, differed considerably from these 25 age-matched ‘typical’ aviremic individuals in four key respects (Fig. 2d–h and Extended Data Table 2). First, according to history from the mother, in four cases, cART had been discontinued completely for 3–10 months, and, in the fifth case, cART adherence was intermittent for 17 months. Second, longitudinal analysis of plasma cART levels showed, after an initial period of adherence, that aviremia was subsequently maintained despite low/undetectable cART levels at most timepoints. Third, four of the five cases were temporarily lost to follow-up at the clinic for 8–17 months. In most cases in South Africa, monthly attendance at a specified clinic is the principal means of accessing the cART prescribed. Finally, despite strong evidence of cART non-adherence, in all five cases this was associated with sustained plasma aviremia, together with an undetectable total HIV DNA load at most timepoints, determined using a digital-droplet polymerase chain reaction (PCR) protocol. By contrast, total HIV DNA loads in the age-matched ‘typical’ aviremic children were, in most cases, detectable at all ages studied (at median age 46 months, median total HIV DNA load 14 copies per million (cpm) peripheral blood mononuclear cells (PBMCs), interquartile range (IQR) 5–74 cpm PBMCs; Supplementary Fig. 1). Moreover, to evaluate HIV-1 DNA further in the five ‘atypical’ individuals, single-genome PCR using different sets of primers previously shown to reliably amplify clade C proviral sequence^24–26^ was attempted; however, we failed to detect any type of HIV-1 amplification products after analyzing 6–12 million PBMCs, isolated 48–61 months after birth, from each of these five infants (Extended Data Table 3).

**Extended Data Fig. 2:**
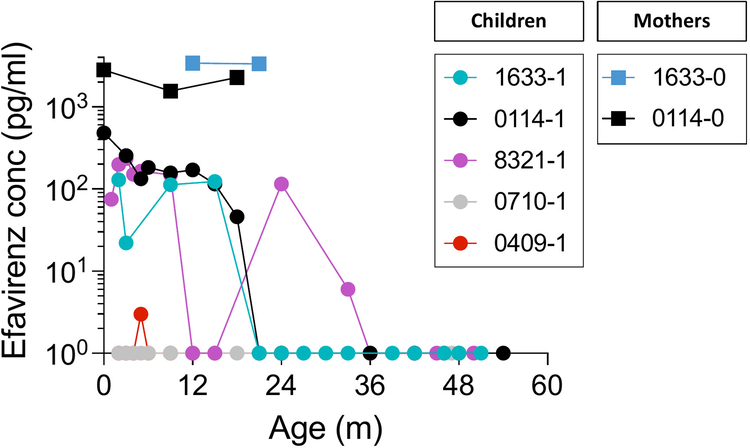
Plasma Efavirenz levels in the 5 ‘atypical’ children and in two of their mothers. The mothers 1633-0 and 0114-0 have the subscript -0, the children have the subscript -1. Three children were breast-fed, 60-1633-1, 80-0114-1 and 60-8321-1; two children, 70-0710-1 and 80-0409-1, were not breast-fed.

Three children within this atypical group were breastfed, suggesting the possibility that maternal cART may have contributed to sustained aviremia in the children^16,27^. However, plasma efavirenz concentrations while breastfed were considerably below the therapeutic range^28^ (1,000–4,000 ng ml^−1^) and less than 5% of those in the maternal plasma (median 2,810 ng ml^−1^; Extended Data Fig. 2).

A further possibility, that these five children may have been administered anti-HIV medications that they were not prescribed, was considered. In one child, 60-1633-1, at the 61-month timepoint, DTG, lamivudine and tenofovir prescribed to the mother were detected in the child’s plasma; however, otherwise, maintenance of aviremia in these children was not explained by access to alternative cART.

### Western blot analysis supports infection in atypical cases

**Extended Data Fig. 3:**
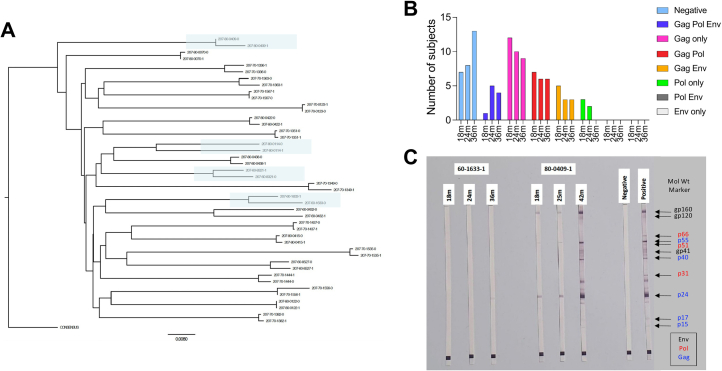
Phylogenetic clustering of mother-child pair *gag* sequences and Western blot testing of plasma samples at 18m, 24m and 36m from children within the study cohort. **a**. A maximum likelihood tree constructed using full-length *gag* sequences shows mother-child clustering of sequences for 22 representative mother-child pairs within the cohort, including 4 of the ‘atypical’ pairs 60-1633-0/1, 80-0114-0/1, 60-8321-0/1, and 80-0409-0/1 (shaded). Maternal sequences carry the suffix -0 and child sequences carry the suffix -1. **b**. Western blot reactivities for 35 children who maintained plasma viral loads at <20c/ml at all timepoints to 36m following suppression. These data include the 3 ‘atypical’ children, 60-1633-1, 80-0114-1 and 70-0710-1, who maintained plasma viral loads at <20c/ml at all timepoints to 36m following suppression. **c**. Western blot results for two of the ‘atypical’ children, 60-1633-1 and 80-0409-1 at the timepoints shown. These data are representative of experiments run in triplicate.

The five children described here maintaining aviremia in the absence of cART more than met the criteria for HIV infection (Extended Data Table 4). Additionally, in four cases, HIV *gag* was amplified from baseline samples of plasma RNA, and mother–child sequences clustered on a phylogenetic tree (Extended Data Fig. 3, panel a). Furthermore, evidence of HIV infection in children arises from detection of antibody responses at ≥18 months of age^29,30^. Before this, anti-HIV antibody might represent maternal antibody crossing the placenta; breast milk–transferred maternal antibodies do not enter the neonatal or infant circulation in humans^31^. However, of 35 children (excluding the atypical cases) who maintained aviremia to ≥36 months without any viral blips or spikes recorded since achieving suppression of viremia by a median of 3 months, only four children tested western blot (WB) negative at 18 months, 24 months and 36 months (Extended Data Fig. 3b).

**Extended Data Fig. 4:**
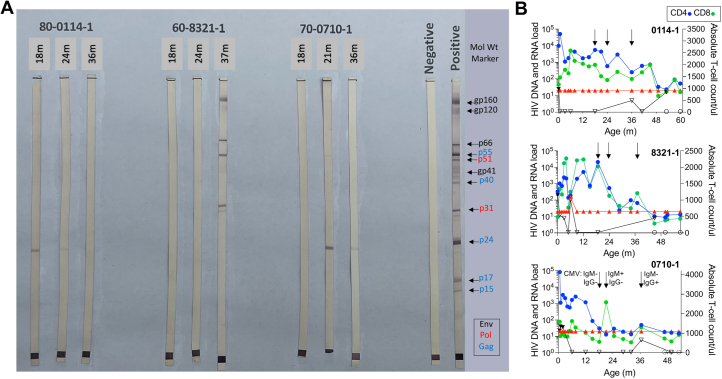
Evidence of HIV infection in the ‘atypical’ male participants from Western Blot testing. **a-b**. Transient anti-HIV antibody detection at age >18m in plasma samples from the 3 ‘atypical’ children whose baseline plasma HIV RNA viral loads were undetectable. These data are representative of experiments run in triplicate. **a**. Western blot testing of plasma samples from 70-0710-1, 60-8321-1 and 80-0114-1 at 18m-36m timepoints. **b**. Plasma HIV RNA viral loads, HIV DNA loads, absolute CD4 count and absolute CD8 count in participants 80-0114-1, 60-8321-1 and 70-0710-1. Symbols as in Fig. 2; blue circles: absolute CD4 counts; green circles: absolute CD8 counts. Arrows show timepoints used for Western blot assays. In 70-0710-1, CMV-specific IgM was undetectable at 18m and 36m and was detectable at 21m; CMV-specific IgG was undetectable at 18m and 21m but detectable at 36m.

Similarly, of the five ‘atypical’ children who maintained aviremia despite cART non-adherence, none was WB negative at 18 months, 24 months and 36 months (Extended Data Figs. 3 and 4). In four cases, there was increased WB reactivity across these timepoints. In the case of 70-0710-1, the absence of any WB bands at 18 months followed by the appearance of p24 and p55 Gag bands at 21 months coincided with cytomegalovirus infection, which strongly activates the immune system and lowers CD4/CD8 ratios^32^ (Extended Data Fig. 4b).

### Type I interferon–resistant, low viral replication capacity viruses are transmitted to females

**Fig. 3:**
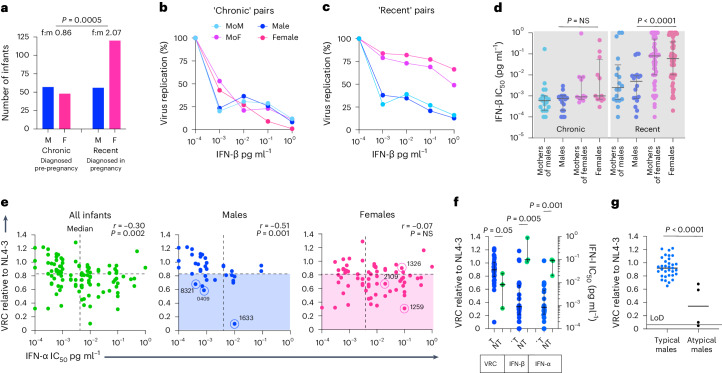
Viruses transmitted to female and male fetuses differ by VRC and IFN-I IC_50_. **a**, Females are more susceptible to in utero transmission when mothers are recently infected but not when they are chronically infected. The *P* value shown was determined using a two-sided Fisher’s exact test. **b**,**c**, Representative examples of viruses transmitted from chronically infected mothers to male and female fetuses (**b**) and from recently infected mothers to male and female fetuses (**c**). **d**, IFN-β IC_50_ values determined for 98 mother–child transmission pairs in the cohort. Data are presented as median and IQR. **e**, Relationship between VRC and IFN-α IC_50_ in viruses transmitted to males and to females. Blue open circles highlight males maintaining aviremia despite cART non-adherence. In the case of 80-0114-1, viral replication was detectable but at levels too low to measure VRC; therefore, IFN-I IC_50_ could not be determined for this child. Red open circles highlight viruses transmitted to the female twin but not to the male twin within three of five sex-discordant twinsets evaluated where, in each case, transmission occurred only in the female twin. Blue shading highlights viruses transmitted to males below the median VRC for all infants; red shading highlights viruses transmitted to females below the median VRC for all infants. The *r* values shown represent Spearman’s rank correlation coefficient, and the *P* values shown were determined using the two-sided Student’s *t-*test. **f**, Comparison of viruses transmitted to male singletons (*n* = 36) with those transmitted to female twins but not transmitted to male twins within three sex-discordant twinsets (that is, *n* = 3). Data are presented as median and IQR. VRC for 80-0114-1 was not included; including 80-0114-1, the *P* value becomes *P* = 0.07. T represents viruses transmitted to male fetuses; NT represents viruses not transmitted to male fetuses. **g**, VRC in ‘typical’ males (aviremia dependent on cART adherence) compared to the four ‘atypical’ males (aviremia not dependent on cART adherence) whose viruses were analyzed. This included VRC for 80-0114-1, which was detectable but too low to quantify. Excluding 80-0114-1, the *P* value becomes *P* = 0.0006. The *P* values shown in **d**, **f** and **g** were determined using the Mann–Whitney *U*-test (two-tailed). F, female; f:m, female to male; LoD, limit of detection; M, male.

Each of the five children described here maintaining aviremia in the absence of cART were male, contrasting with the cohort as a whole (60% female, *P* = 0.01, Fisher’s exact test). We, therefore, considered possible mechanisms that might account for a higher rate of sustained cART-free aviremia in males. We previously reported^33^ that female fetal susceptibility to in utero transmission is higher than that of male fetuses only in the setting of recently infected mothers. In this cohort, we found a female:male ratio of 2.07 in the setting of recently infected mothers and a female:male ratio of 0.86 in the setting of chronic maternal infection (*P* = 0.0005; Fig. 3a). In adult-to-adult transmission^34–36^, the founder virus is highly type I interferon (IFN-I) resistant, but, over 6–12 months, the circulating viral quasi-species in the recipient become increasingly IFN-I sensitive. We, therefore, hypothesized, first, that fetuses born to chronically infected mothers are exposed in utero to IFN-I-sensitive viruses, whereas fetuses born to recently infected mothers are exposed principally to IFN-I-resistant viruses; and, second, that female fetuses would be more susceptible to IFN-I-resistant viruses because IFN-I production is higher in innate immune cells from female children, adolescents and adults than from males^37–40^.

**Extended Data Fig. 5:**
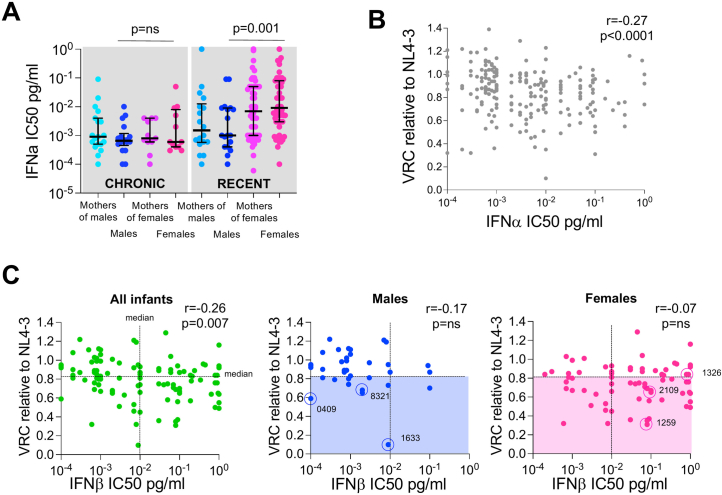
Inverse correlation between VRC and IFN-I resistance in study cohort. **a**. IFNa IC50 values determined for 98 mother-child pairs in the cohort. P values were determined using the two-tailed Mann-Whitney U test. **b**. Inverse correlation overall in study cohort between VRC and IFNa IC50. Data are presented as median and interquartile range. The P value was determined using the two-tailed Student’s *t-*test. **c**. Relationship between VRC and IFNa IC50 in viruses transmitted to males and to females. Blue open circles highlight males maintaining aviraemia despite cART non-adherence. In the case of 80-0114-1, viral replication was detectable but at levels too low to measure VRC; therefore IFN-I IC50 could not be determined for this child. Red open circles highlight viruses transmitted to the female twin but not to the male twin within 3 of 5 sex-discordant twinsets evaluated where, in each case, transmission only occurred in the female twin. Blue shading highlights viruses transmitted to males below the median VRC for all infants; red sharing highlights viruses transmitted to females below the median VRC for all infants. r values represent Spearman’s rank correlation coefficient. The P values were determined using the two-tailed Student’s *t-*test.

To determine the IFN-I sensitivity of transmitted viruses, we developed a high-throughput reporter assay using the IFN-I-sensitive U87 human glioblastoma cell line modified to express CXCR4 and CCR5 and stably transduced with a lentivirus vector to express secreted nanoluciferase in response to HIV-1 infection and Tat expression. We generated Gag-Pro-NL4-3 chimeric viruses from 98 mother–child pairs to determine IFN-α and IFN-β half-maximal inhibitory concentration (IC_50_) values (Fig. 3b,c) and, at the same time, determine the viral replicative capacity (VRC) of transmitted viruses. As hypothesized, viruses transmitted to female fetuses were more IFN-I resistant than those transmitted to males but only in the setting of recent maternal infection (*P* = 0.001 and *P* < 0.0001 for IFN-α and IFN-β, respectively; Fig. 3d and Extended Data Fig. 5, panel a).

**Extended Data Fig. 6:**
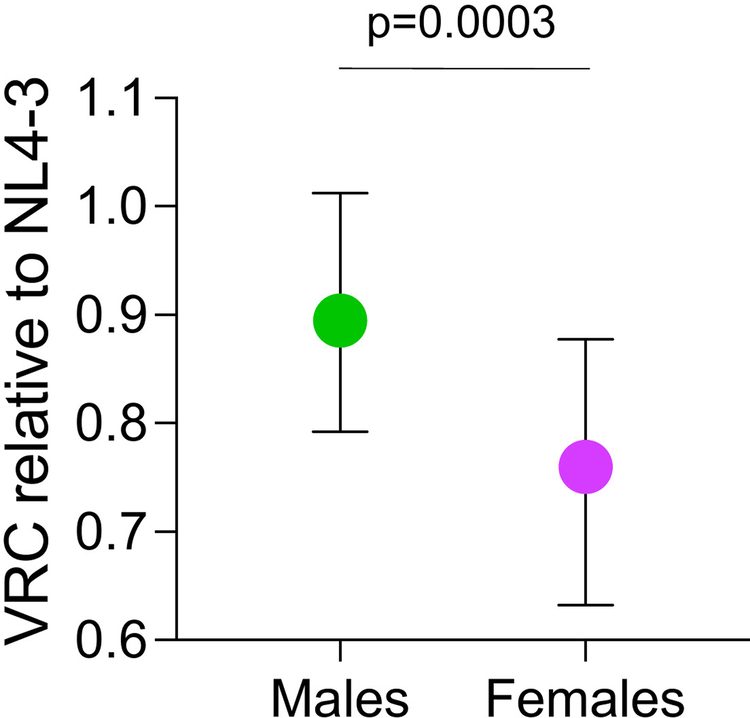
Baseline viral replicative capacities in male and female infants. Males n = 36, females n = 68. Data are presented as median and interquartile range. P value determined using the two-sided Mann-Whitney U test.

We next determined the VRC of the same Gag-Pro-NL4-3 viruses. The viruses transmitted to female fetuses overall had lower VRC than those transmitted to male fetuses (*P* = 0.0003; Extended Data Fig. 6). Viral mutations selected to confer IFN-I resistance may, like other immune escape variants^41–44^, incur a cost to VRC and, hence, may be selected only in females where strong IFN-I innate immune responses impose sufficient selection pressure on the virus. Notably, we observed a negative correlation between IFN-I IC_50_ and VRC in the entire cohort (for IFN-α: infants, *r* = −0.30, *P* = 0.002, Fig. 3e; infants and mothers, *r* = −0.27, *P* < 0.0001, Extended Data Fig. 5b) and a stronger correlation among males than females (IFN-α: for males, *r* = −0.51, *P* = 0.001; for females, *r* = −0.07, *P* not significant (NS); Fig. 3e and Extended Data Fig. 5c).

To further characterize the differences between the viruses transmitted to female fetuses and not to male fetuses, we studied the five sex-discordant twinsets in the cohort in which transmission had arisen in only one twin. In our cohort, only the female twin acquired HIV in all five twinsets, similar to previously published twin studies^45^. Samples were available from three of the five twinsets. Compared to the transmitted viruses in male singletons, the three viruses transmitted to the female but not to the male twin (red-circled in Fig. 3e and Extended Data Fig. 5c) had somewhat lower VRCs (*P* = 0.05) but substantially higher IFN-I IC_50_ values (*P* = 0.001 and *P* = 0.005 for IFN-α and IFN-β, respectively; Fig. 3f).

Lastly, in four of the five ‘atypical’ males described, the VRC of the viruses transmitted was substantially lower than that of the viruses transmitted to other males in the cohort (Fig. 3g; *P* < 0.0001 (in one instance, viral replication was detectable but at levels too low to quantify)). We propose that these collective findings are consistent with the hypothesis that in utero transmission followed by sustained productive infection in male infants is dependent upon the transmission of high-replication-capacity, IFN-I-sensitive viruses.

### Selection of transmitted viruses by fetal immunity

**Fig. 4:**
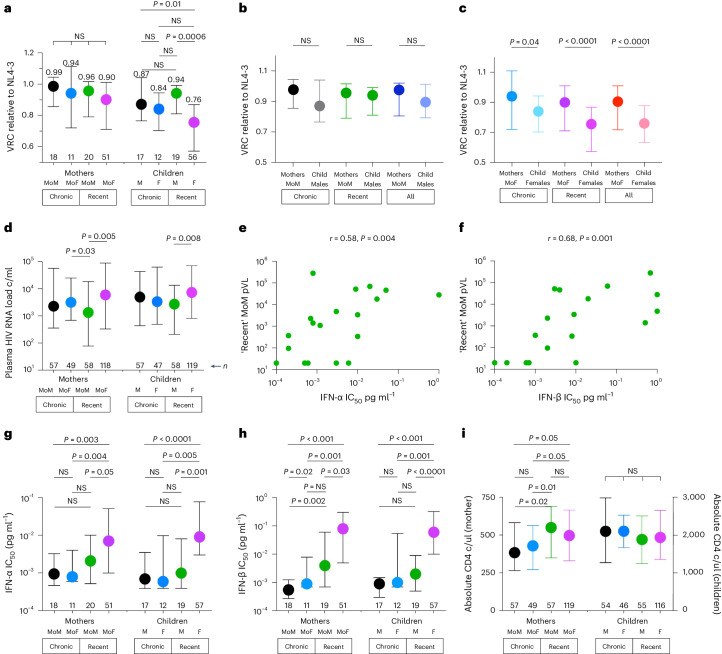
pVL, VRC, IFN-I IC_50_ values and absolute CD4 counts for the mother–child pairs within the Ucwaningo cohort. In **a**, **d** and **g**–**i**, mothers and infants are subdivided according to timing of maternal infection—chronically infected (‘Chronic’) and recently infected (‘Recent’)—and according to the sex of the infant: MoM, MoF, male (M) infants and female (F) infants. In all cases, data are from the time of enrollment/baseline. **a**, VRC relative to NL4-3. Median VRC values are shown. **b**,**c**, Comparison of VRC between MoM and males (**b**) and between MoF and females (**c**). **d**, pVLs. **e**,**f**, Correlation between pVL from recently infected MoM and IFN-I IC_50_ (IFN-α and IFN-β, **e** and **f**, respectively). *r* values shown are Spearman’s rank correlation coefficients. *P* values shown were determined by the two-sided Student’s *t*-test. **g**, IFN-α IC_50_. **h**, IFN-β IC_50_. **i**, Absolute CD4 counts. In **a**–**d** and **g**–**i**, data are presented as median and IQR. Other than for the tests applied in **e** and **f**, the *P* values shown in this figure were determined using two-tailed Mann–Whitney *U*-tests or, where comparisons were made between MoM and males or between MoF and females, using the two-tailed Wilcoxon matched-pairs signed-rank test. c/ml, copies per milliliter; c/ul, cells per microliter.

To further investigate whether there is evidence of selection imposed by the fetal immune system, not only on whether transmission arises but also on the quality of viruses transmitted from the mother, we compared VRCs, IFN-α and IFN-β IC_50_ values, pVL, and absolute CD4 counts for the mothers of males (MoM), for the mothers of females (MoF), and for male and female infants, segregated by timing of maternal infection (Fig. 4). We observed that, although VRC (‘fitness’) does not differ between the viruses circulating in recently infected transmitting MoM and MoF (Fig. 4a), the viruses transmitted to female fetuses are of lower ‘fitness’ than those transmitted to male fetuses (*P* = 0.0006; Fig. 4a) and are also of substantially lower ‘fitness’ than those circulating in the MoF (*P* < 0.0001; Fig. 4c). By contrast, no difference was observed between the VRC/‘fitness’ of viruses circulating in MoM versus those transmitted to male fetuses (Fig. 4b).

Finally, we observed that pVL of recently infected MoM is lower than that of MoF (Fig. 4d). As expected, pVL in these mothers was strongly correlated with IFN-I IC_50_ (*r* = 0.58 and *r* = 0.68 for IFN-α and IFN-β, respectively; *P* = 0.004 and *P* = 0.001; Fig. 4b,c). Using a univariate linear regression model to determine the impact of IFN-α IC_50_ and IFNβ IC_50_ on pVL in the mothers (Supplementary Table 1), we note that the difference in IFN-I IC_50_ between transmitting MoM and transmitting MoF corresponds to a pVL difference of between 0.43 log_10_ and 0.78 log_10_ and that this, therefore, accounts fully for the observed 0.65 log_10_ difference in pVL between MoM and MoF.

## Discussion

These observational studies of a cohort of 284 very-early-cART-treated children after in utero HIV infection identify a distinct group of five ‘atypical’ males maintaining aviremia in the absence of cART. Cases of post-treatment control after pediatric infection are rare^46–49^ but have provided important evidence that functional cure or remission may be achievable in this setting. However, the conclusions that can be drawn from these and from the current study are limited by the small number of individuals described. Also, the cases previously described were quite dissimilar. In particular, the clade of virus, timing of vertical transmission and of cART initiation, age at viral suppression and CD4 nadir all differed from case to case. In the current study, the five atypical children are all male, whereas the cohort as a whole is 60% female. The four transmitted viruses analyzed had significantly lower VRC compared to those transmitted to ‘typical’ males. In two cases (60-1633-1 and 80-0114-1), VRC was exceptionally low. Of note, in both of these instances, the mothers seroconverted in the latter weeks of pregnancy, potentially increasing levels of immune activation in the fetus via the high levels of immune activation in the mother during acute infection. The risk of in utero vertical transmission in the absence of ART is increased three-fold by acute maternal infection^9^. These findings recall a report of spontaneous HIV clearance in a male infant after likely maternal seroconversion in pregnancy in the pre-cART era^50^. Together, these observations prompt the hypothesis that in utero transmission of high-replicative-capacity viruses may be needed to sustain HIV infection in males, whereas, in females, who have higher levels of immune activation both antenatally and postnatally^33,37^, low-replicative-capacity/IFN-I-resistant viruses can propagate efficiently.

Previous studies in adults showed that the replication capacity of the transmitted virus influences pro-viral DNA load, levels of immune activation and disease outcome in the recipient^14^. Thus, the potential for cure/remission may initially be highest in those male infants who are infected with low-replicative-capacity viruses, especially if these are IFN-I sensitive and, therefore, more readily contained by innate immunity. However, beyond approximately 2 years of age, immune control of HIV is superior in females^17,18,51,52^, and it is possible that the fine balance that exists between the disadvantages of a more activated immune system—in this case, greater susceptibility to in utero infection—and the advantages of more effective anti-viral immunity may favor females infected with low-replicative-capacity viruses in achieving post-treatment control later in childhood. Of note, the NK cell responses implicated in post-treatment control^1,4^ and in reducing genome-intact HIV-1 DNA levels after ART initiation in neonates^24,53^ have recently been shown to be stronger in female mice, despite lower absolute numbers of NK cells in females^54^.

It is important to highlight limitations of this study. First, as mentioned above, the numbers of children described here achieving cART-free aviremia are small, and further studies are needed. Second, without a documented anti-retroviral therapy interruption (ATI), it is possible that cART was taken intermittently or that transient viral rebounds occurred between clinic visits and during periods of loss to follow-up. To that end, ATI studies were recently initiated in a selected subset of this cohort. To date, three of the five ‘atypical’ children described in this study have remained aviremic for more than 12 months during the ATI. In one case, 60-8321-1, cART has not been restarted so far for more than 30 months, after an additional period of 8 months of cART discontinuation in the child according to history. In adults, the median time to viral rebound after ATI is 3 weeks, 5% remaining suppressed 12 weeks after ATI^55–57^. ATI studies in children are rarer, with median time to viral rebound reportedly between 2 weeks and 8 weeks and 94–100% rebounding by 6 months^58–61^. Together, these data suggest that the five children reported here are highly unusual in maintaining aviremia for the duration of time described here.

A further caveat is the use of plasma cART testing to assess adherence, in conjunction with the history from the caregiver, pill counting and pharmacy records (Extended Data Table 2 and Supplementary Fig. 2). The measurement of cART levels in the plasma at clinic visit does not necessarily reflect cART adherence for the entire period of time between clinic visits. Nonetheless, even for a single sample taken at the 12-month timepoint, there is a strong correlation between cART levels and viremia. This is consistent with previous observations in this cohort that viral rebound is rarely explained by cART resistance and almost always by cART non-adherence^20^. Furthermore, these findings presented here indicate that aviremia in most children is highly cART adherence dependent.

An additional caveat is our use of Gag-Pro/NL4-3 chimeric viruses as opposed to full-length virus isolates to assess IFN-I sensitivity and VRC in this cohort. The reasons for using this approach are three-fold. First, the Gag-Pro/NL4-3 chimeric approach is high throughput, enabling the quasi-species of many individuals (98 mother–child pairs here) to be rapidly evaluated, allowing sex differences to be analyzed, which the substantially more labor-intensive approach using whole virus isolates precludes^34–36,62,63^. Second, our approach involves unbiased polyclonal amplification of the entire swarm of viruses present in the plasma sample, cloned into the NL4-3 backbone as a swarm of Gag-Pro/NL4-3 chimeric viruses. The chimeric virus swarm was shown in numerous studies^13,14,33,64–75^ to be highly representative of the circulating quasi-species. The alternative, generating full-length viral isolates via single-genome amplification, is problematic because it is not at all certain whether each clone is representative of the viral swarm. Confirmation via deep sequencing of full-length virus is not generally feasible in the setting of low pVL levels that are fairly common in the children being studied here. Finally, although variation outside of Gag-Pro including Env can also affect IFN-I sensitivity, the biological relevance of the Gag-Pro/NL4-3 virus has been thoroughly addressed^3,13,14,64–75^. The replication of these recombinant viruses is strongly correlated with that of full-length virus isolates and recapitulates clade-specific differences in replication^64^. Furthermore, the Gag-Pro region is known to bind to well-characterized IFN-I-stimulated restriction factors, such as TRIM5α and MX2 (refs. ^76,77^). Analysis of this relatively highly conserved part of the proteome in longitudinal studies of viruses harbored by individuals from acute into chronic infection should, in the future, facilitate the definition of specific variants that affect IFN-I sensitivity.

Next steps in evaluating the ‘atypical’ males described here would first include ATI studies to identify additional cases of very-early-cART-treated children who can maintain cART-free aviremia. To date, we have depended on chance findings of aviremia in children after prolonged cART discontinuation. However, the current study and reports from the P1115 trial^78^ suggest that prospective ATI studies will reveal additional cases that, together, would facilitate identification of genetic, virological and immune factors contributing to post-treatment control in children. In addition, as these children progress to adolescence, it may become feasible to undertake, via leukapheresis, more comprehensive analyses of the viral reservoir.

In conclusion, irrespective of how early cART is initiated, maintenance of aviremia in children is highly cART dependent, and, in most cases, cure/remission strategies will depend on interventions additional to early cART initiation. However, a small subset of early-cART-treated children exists that may achieve post-treatment control. The factors identified in this cohort that associate with viral control include male sex and the transmission of low-replicative-capacity, IFN-I-sensitive virus. Most adult studies of ATI have focused on males from resource-rich settings, and initial indications are that differences may exist between the sexes with respect to post-treatment control in resource-limited settings^79^. Further studies are needed to determine the impact of sex and other genetic, viral and immune factors on post-treatment control at different ages as well as the additional interventions that will accelerate functional cure in early-cART-treated children and identify the subsets of children that are most likely to achieve it.

## Methods

### Study participants

The Ucwaningo Lwabantwana (meaning ‘Learning from Children’) is a cohort of 284 in utero–infected children enrolled and followed in KwaZulu-Natal, South Africa, from 2015 to 2023. All infants were tested at birth via the SoC dried blood spot total nucleic acid PCR (COBAS AmpliPrep/COBAS TaqMan HIV-1 Qualitative PCR v2, Roche Molecular Diagnostics) that was run in a central laboratory. If the result of this test was positive or indeterminate, a confirmatory or repeat test, respectively, was undertaken at approximately 7 days of age. All children born to mothers LWH received ART in the delivery room within minutes of birth (either NVP alone or AZT plus NVP, according to local guidelines). Infants of mothers at high risk of in utero HIV transmission were also tested for HIV-1 as soon as possible after birth using PoC testing (in addition to the SoC testing) to detect total nucleic acid via PCR on whole blood (GeneXpert Qualitative HIV-1 PCR, Cepheid): 84 infants in the cohort were diagnosed via PoC testing, and the remaining 200 were diagnosed via SoC testing. In all 284 enrollees, confirmed diagnosis required two separate samples detecting HIV nucleic acid. Baseline data were collected at a median of 1.0 (IQR 0.9–1.8) day of age and 11 (IQR 9–14) days of age from the PoC-diagnosed and SoC-diagnosed infants, respectively. Initial cART for infants with confirmed HIV infection comprised twice-daily NVP, AZT and lamivudine (3TC) as per local guidelines. This regimen was switched to RTV-boosted LPV, 3TC and abacavir (ABC) at 42 weeks corrected gestational age or at 1 month of age. Mother and infant follow-up occurred monthly for 6 months and then every 3 months. At each visit, blood was drawn for CD4^+^ T cell quantification, pVL (HIV-1 RNA PCR, NucliSens) and storage of PBMCs and plasma. This study was approved by the Biomedical Research Ethics Committee of the University of KwaZulu-Natal and the Oxfordshire Research Ethics Committee. Written informed consent for the infant’s and mother’s participation in the study was obtained from the mother or the infant’s legal guardian.

### Viral RNA isolation and nested RT–PCR amplification of *gag-protease* from plasma

Viral RNA was isolated from plasma by use of a QIAamp Viral RNA Mini Kit (Qiagen). The *Gag-Protease* region was amplified by PCR with reverse transcription (RT–PCR) from plasma HIV-1 RNA using a SuperScript III One-Step Reverse Transcriptase Kit (Invitrogen) and the following *Gag-protease*-specific primers: 2cRx, 5′ CAC TGC TTA AGC CTC AAT AAA GCT TGC C 3′ (HXB2 nucleotides 512–539), and 623Fi, 5′ TTT AAC CCT GCT GGG TGT GGT ATT CCT 3′ (nucleotides 2,851–2,825). Second-round PCR was performed using 100-mer primers that completely matched the pNL4-3 sequence using Takara EX Taq DNA Polymerase, Hot Start version (Takara Bio). One hundred microliters of reaction mixture was composed of 10 µl of 10× EX Taq Buffer, 4 µl of deoxynucleoside triphosphate mix (2.5 mM each), 6 µl of 10 µM forward primer, *Gag-Pro* F (GAC TCG GCT TGC TGA AGC GCG CAC GGC AAG AGG CGA GGG GCG GCG ACT GGT GAG TAC GCC AAA AAT TTT GAC TAG CGG AGG CTA GAA GGA GAG AGA TGG G, 695–794) and reverse primer, *Gag-Pro* R (GGC CCA ATT TTT GAA ATT TTT CCT TCC TTT TCC ATT TCT GTA CAA ATT TCT ACT AAT GCT TTT ATT TTT TCT TCT GTC AAT GGC CAT TGT TTA ACT TTT G, 2,646–2,547), 0.5 µl of enzyme and 2 µl of first-round PCR product and DNase-RNase-free water. Thermal cycler conditions were as follows: 94 °C for 2 min followed by 40 cycles of 94 °C for 30 s, 60 °C for 30 s and 72 °C for 2 min and then followed by 7 min at 72 °C. PCR products were purified using a QIAquick PCR Purification Kit (Qiagen) according to the manufacturer’s instructions.

### Generation of recombinant *Gag-Protease* viruses

A deleted version of pNL4-3 was constructed that lacks the entire *Gag* and *Protease* region (Stratagene QuikChange XL kit), replacing this region with a BstE II (New England Biolabs) restriction site at the 5′ end of *Gag* and at the 3′ end of *protease*. To generate recombinant viruses, 10 µg of BstEII-linearized plasmid plus 50 µl of the second-round amplicon (approximately 2.5 µg) were mixed with 2 × 10^6^ cells of a Tat-driven green fluorescent protein (GFP) reporter T cell line (GXR 25 cells) in 800 µl of R10 medium (RPMI 1640 medium containing 10% FCS, 2 mM L-glutamine, 100 U ml^−1^ penicillin and 100 µg ml^−1^ streptomycin) and transfected by electroporation using a Bio-Rad Gene Pulser II instrument (300 V and 500 uF). After transfection, cells were rested for 45 min at room temperature, transferred to T25 culture flasks in 5 ml of warm R10 and fed with 5 ml of R10 on day 4. GFP expression was monitored by flow cytometry (FACSCalibur, BD Biosciences), and, once GFP expression reached more than 30% among viable cells, supernatants containing the recombinant viruses were harvested and aliquots stored at −80 °C.

### VRC assays

The replication capacity of each chimera was determined by infection of GXR cells at a low multiplicity of infection of 0.003, following the method of Miura et al.^65^. The mean slope of exponential growth from day 2 to day 7 was calculated using the semi-log method in Excel. This was divided by the slope of growth of the wild-type NL4-3 control included in each assay to generate a normalized measure of replication capacity. Replication assays were performed in triplicate, and results were averaged. These VRC determinations were undertaken entirely blinded to the identity of the study subject.

### Single-genome amplification of viral RNA

For single-genome amplification of the gag-protease genes, RNA was endpoint diluted in 96-well plates such that fewer than 29 PCRs yielded an amplification product. According to a Poisson distribution, the cDNA dilution that yields PCR products in no more than 30% of wells contains one amplifiable cDNA template per positive PCR more than 80% of the time^80^. RT–PCR and second-round amplification were carried out as described above. PCR products were visualized by agarose gel electrophoresis under UV light using a Gel Doc 2000 (Bio-Rad). All products derived from cDNA dilutions yielding less than 30% PCR positivity were purified using a QIAquick PCR Purification Kit (Qiagen) and sequenced.

### Viral RNA sequencing and phylogenetic analysis

Population sequencing was undertaken using the BigDye Ready Reaction Terminator Mix (V3) (Department of Zoology, University of Oxford) using *gag-protease* sequencing primers SQ2FC (CTT CAG ACA GGA ACA GAG GA), GF100-1817.18 (TAG AAG AAA TGA TGA CAG), gf2331 (GGA GCA GAT GAT ACA GTA TT), SQ16RC (CTT GTC TAG GGC TTC CTT GGT), GAS4R (GGT TCT CTC ATC TGG CCT GG), pan1dRx (CAA CAA GGT TTC TGT CAT CC), GR1981 (CCT TGC CAC AGT TGA AAC ATT T) and gr2536 (CAG CCA AGC TGA GTC AA). Sequence data were analyzed using Sequencher version 4.8 (Gene Codes Corporation). Nucleotides for each gene were aligned manually in Se-Al version 2.0a11. Maximum-likelihood phylogenetic trees were generated using PHYm131 (https://www.hiv.lanl.gov) and visualized using FigTree (http://tree.bio.ed.ac.uk/software/figtee/) version 1.2.2.

### IFN-I sensitivity assays using the U87 MG/D1406 cell line

The U87 CD4^+^CCR5^+^ cells were obtained from the National Institutes of Health (NIH) HIV Reagent Program (cat. no. 4035), contributed by HongKui Deng and Dan Littman^81^. The U87/D1406 reporter cell line was generated by transduction with a lentivirus vector (K5534) comprising secreted nano-luciferase (snLuc), monomeric enhanced green fluorescence protein (EGFP) and puromycin (puro), wherein expression of these genes was placed under control of an HIV-1 long terminal repeat (LTR) (LTR-puro.T2A.EGFP-IRES-snLuc-LTR). The parental U87 CD4^+^CCR5^+^ cells are resistant to G418 and puro. Therefore, after transduction with the K5534 lentivector, the cells were FACS sorted to isolate a population of cells exhibiting nominal GFP expression, thereby reducing the background level of snLuc expression from uninfected cells. The cells were further modified by transduction with a lentivirus vector (K5600) comprising the human CXCR4 co-receptor and the blasticidin resistance gene (BSD) that were placed under transcriptional control of the human EF1αpromoter (EF1α-CXCR4-IRES-BSD). The U87/D1406 reporter cells are sensitive to infection by either R5 or X4 strains of HIV-1. Infected cells express EGFP and snLuc that may be sampled directly from culture supernatants and analyzed to quantify HIV-1 infection and inhibition thereof.

To determine IFN-I concentrations required to inhibit virus replication to 50% (IC_50_), U87-MG cells were left untreated or cultured in the presence of increasing amounts of IFN (0.0001 pg ml^−1^ to 1 pg ml^−1^), infected with equal amounts of patient-specific gag-pro chimeric virus (multiplicity of infection 0.03) and cultured for 6 days (IFN-α-2α BioVision 4594-100 lot 6HO6L45940 and IFN-β BioVision 4860-50 lot 40460). IFN-containing medium was replenished every 24 h. Viral infectivity was measured using a luminescent reporter (Nano-Glo Luciferase Assay System, Promega) and quantified on a GloMax 96 microplate luminometer (Promega). Viral replication was plotted for each IFN concentration as the percentage of viral growth in the absence of IFN.

### pVL

Plasma HIV-1 RNA viral load measurement for the Ucwaningo Lwabantwana cohort (2015–2023) was undertaken using the BioMérieux NucliSens Version 2.0 Easy Q/Easy Mag (NucliSens version 2.0) assay (dynamic range, 20–10 million HIV RNA copies per milliliter). Plasma HIV-1 RNA viral load measurement for the Paediatric Early HAART and Structured Treatment Interruption Study (PEHSS) cohort (2002–2005) was undertaken using the Roche Amplicor version 1.5 assay, with a lower limit of detection of 50 RNA copies per milliliter.

### HIV-1 DNA analysis

Total HIV-1 DNA levels in the pediatric cohort were quantified using droplet-digital PCR (Bio-Rad) starting from 1 × 10^6^ PBMCs. Samples were screened with three different primer/probe sets, two annealing to the 5′*LTR* and *gag* conserved regions of HIV-1 genome^82^ and an additional *int-degenerated* primer/probe set adapted to clade C (iSCA-int Forward Degenerate TTTGGAAAGGACCAGCMAA, iSCA-int Reverse TGGAAAACAGATGGCAGG, iSCA-int Probe AAAGGTGAAGGGGCAGTAGTAATACA). Input cell number was verified by quantifying the amount of genomic *RRP30* gene, in a parallel droplet-digital PCR assay, and this value was used to infer the limit of detection for each sample. Viral DNA was further evaluated using PCR protocols designed to amplify near full-length, gag or pol clade C HIV-1 sequences, as described in Extended Data Table 3. Primers and reaction conditions for near full-length amplification were as described by Garcia-Broncano et al.^24^, Lee et al.^25^ and Dufour et al.^83^; for the latter experiments, the reverse primer of the second-round PCR was adapted to 5′-CTAGTTACCAGAGTCACACAACAGACG-3′. Primers and reaction conditions for amplification of gag were as described by Koofhethile et al.^26^; for the amplification of HIV-1 pol, the following primer pairs were used: forward primer 5′-GAAGGGCACATAGCCAGAAATTGCAGGG-3′, reverse primer 5′-GCTCCTGCTATGGGTTCTTTCTCCAGCTGG-3′; second-round nested PCR forward primer 5′-CCTAGGAAAAAAGGCTGTTGGAAATGTGG-3′, reverse primer 5′-CAAACTCCCACTCAGGAATCCA-3′.

### Determination of plasma cART levels

We focused cross-sectionally on children enrolled in the first 30 months of the study (*n* = 73, median age 12 months, IQR 12–12 months) and, additionally, in longitudinal analyses, on the children aged ≥48 months in the cohort that had maintained aviremia consistently without any viral blips or spikes (*n* = 25). At baseline, all infants were initiated on AZT/3TC/NVP, which was switched at 1 month to ABC/3TC/LPV/RTV. We determined the levels of all 13 ART drugs prescribed in South Africa but focused on LPV because its plasma level is directly related to anti-viral activity; also, its half-life is longer than that of the nucleoside reverse transcriptase inhibitors, such as AZT, and, therefore, is less affected by variation in sampling time after dosing. Blood samples were taken in clinic within 2–4 h of the child being administered with cART, according to maternal history. Children were treated with the approved formulation of liquid. Plasma samples were analyzed using LC–MS/MS. The LC–MS/MS method for the simultaneous detection and quantitation of 13 anti-retroviral drugs was developed and validated according to US Food and Drug Administration guidelines for bioanalytical analysis^21,84^. The quantitative concentration range was 150–6,000 ng ml^−1^ for LPV. For values below 150 ng ml^−1^, the concentration of drug can be determined with a degree of certainty if the signal-to-noise ratio for that particular sample is sufficiently high. Here, we used a signal-to-noise ratio of ≥3, as is widely accepted^85^, for all values shown lower than 150 ng ml^−1^ but higher than 10 ng ml^−1^. A 10× dilution using drug-free plasma was used to quantify drug levels in samples with drug levels above the upper limit of quantification. Sample analysis was performed using an Agilent 1200 series high-performance liquid chromatography (HPLC) system coupled to SCIEX QTRAP 5500 triple quadrupole mass spectrometer equipped with an electrospray ionization TurboIonSpray source. Calibration standards and quality control samples were prepared using drug-free, donor human plasma. The anti-retroviral drug analytes were extracted from standard, quality control and clinical samples using a protein precipitation method. The extracted analytes were chromatographically separated on an Agilent ZORBAX Eclipse Plus C18 (2.1 × 50 mm, 3.5 µm) HPLC column using gradient elution. The aqueous mobile phase A consisted of water with 0.1% formic acid, and mobile phase B consisted of acetonitrile with 0.1% formic acid. The HPLC column oven temperature was set at 40 °C; a sample volume of 2 µl was injected; and the flow rate was set to 0.2 ml min^−1^. Mass spectrometry data acquisition was performed using polarity switching—for the simultaneous analyte ion selection and detection in positive and negative modes. Analysis was performed using multiple reaction monitoring at unit resolution, in positive scan mode, set to detect parent [M + H]^+^ → product ion transitions for LPV; *m/z* 629.2 → *m/z* 120.6 and *m/z* 155.2. Data were collected and quantitated using Analyst software, version 1.6.2.

### Statistical analysis

In scatter plots, median values and IQRs are indicated. Comparisons were performed using Fisher’s exact test for categorical variables and Student’s *t*-test or Mann–Whitney *U*-test for continuous variables. Maintenance of viral suppression was calculated using Kaplan–Meier analysis, and different groups were compared using the log-rank test. Univariate linear regression analyses were performed with log_10_ (MoM pVL) as response and either log_10_ (IFN-α IC_50_) or log_10_ (IFN-β IC_50_) as covariate, respectively. In addition, multivariate regression models were applied with log_10_ (child pVL) as the response and several covariates (DTG, age at enrollment (AaE), sex and the interaction between AaE and sex). All *P* values in these regression models were two-sided. All calculations and graphs were performed using R software^86^ and GraphPad Prism version 7.

### Reporting summary

Further information on research design is available in the Nature Portfolio Reporting Summary linked to this article.

## Online content

Any methods, additional references, Nature Portfolio reporting summaries, source data, extended data, supplementary information, acknowledgements, peer review information; details of author contributions and competing interests; and statements of data and code availability are available at 10.1038/s41591-024-03105-4.

## Supplementary information

Supplementary Information Supplementary Figs. 1 and 2 and Table 1.

Reporting Summary

## Figures and Tables

**Extended Data Table 1:**
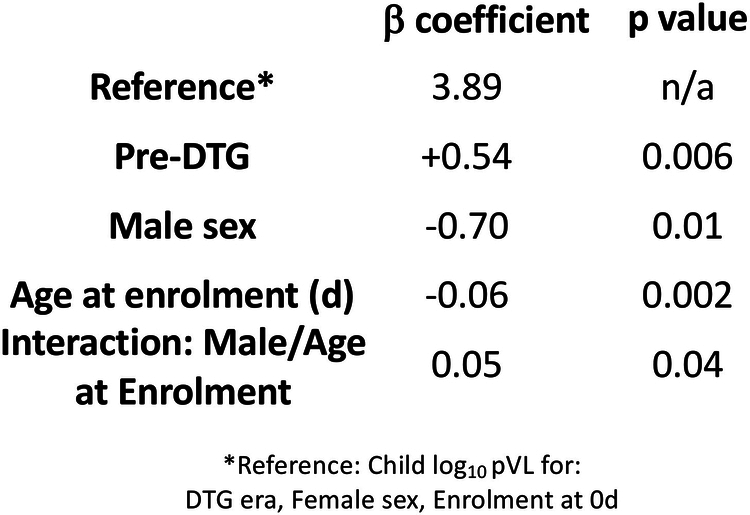
A multivariate regression model to determine the impact on child pVL at baseline of the DTG era, child AaE (days), sex of child and the interaction between sex of child and AeE

The model was applied with log_10_ (child pVL) as the response and DTG, AaE, sex of child and the interaction between AaE and sex of child as the covariates. All *P* values in this regression model were determined using two-sided Studentʼs *t*-tests.

**Extended Data Table 2:**
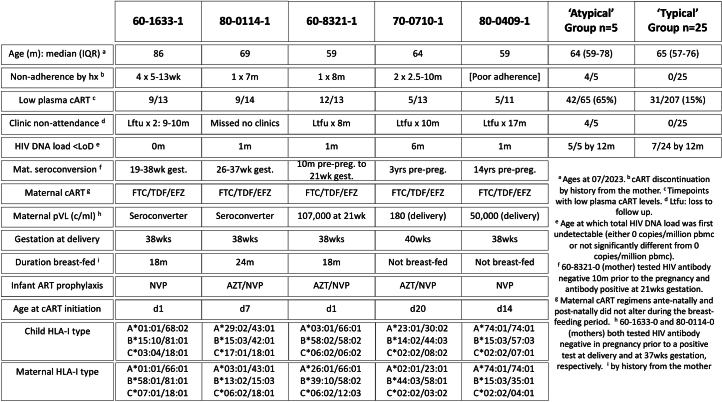
Summary of relevant clinical features of the atypical group of five children compared to the ‘typical’ group of age-matched children

**Extended Data Table 3:**
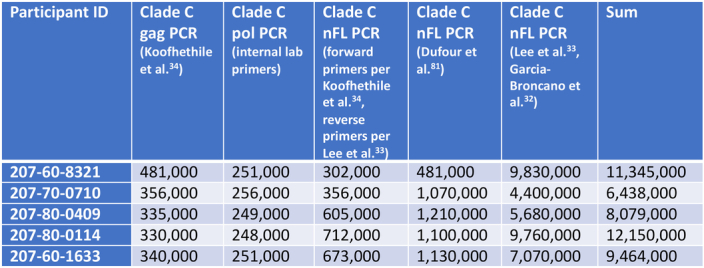
Summary of PCR reactions for analyzing HIV-1 DNA by single-genome amplification

Numbers of PBMCs subjected to each type of PCR reaction are indicated. nFL, near full-length.

**Extended Data Table 4:**
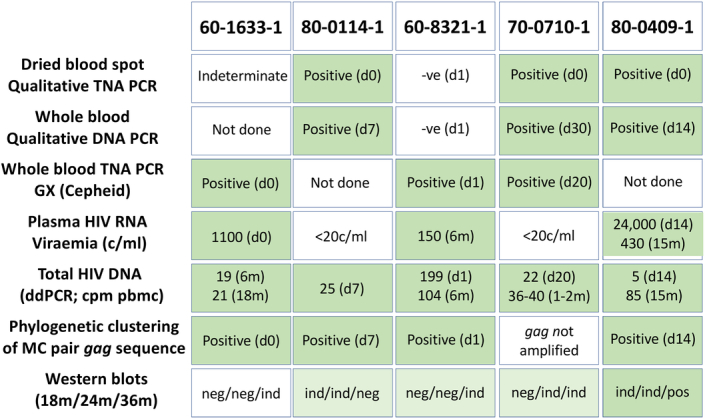
Tests undertaken that determined HIV infection status in the five ‘atypical’ children maintaining aviremia despite cART non-adherence

## Data Availability

There are no restrictions on the availability of materials or information relating to this manuscript.

## Code availability

The code is not available.
