# Author Correction: Sustained aviremia despite anti-retroviral therapy non-adherence in male children after in utero HIV transmission

**DOI:** 10.1038/s41591-025-03555-4

**Published:** 2025-02-05

**Authors:** Nomonde Bengu, Gabriela Cromhout, Emily Adland, Katya Govender, Nicholas Herbert, Nicholas Lim, Rowena Fillis, Kenneth Sprenger, Vinicius Vieira, Samantha Kannie, Jeroen van Lobenstein, Kogielambal Chinniah, Constant Kapongo, Roopesh Bhoola, Malini Krishna, Noxolo Mchunu, Giuseppe Rubens Pascucci, Nicola Cotugno, Paolo Palma, Alfredo Tagarro, Pablo Rojo, Julia Roider, Maria C. Garcia-Guerrero, Christina Ochsenbauer, Andreas Groll, Kavidha Reddy, Carlo Giaquinto, Paolo Rossi, Seohyun Hong, Krista Dong, M. Azim Ansari, Maria C. Puertas, Thumbi Ndung’u, Edmund Capparelli, Mathias Lichterfeld, Javier Martinez-Picado, John C. Kappes, Moherndran Archary, Philip Goulder

**Affiliations:** 1 Queen Nandi Regional Hospital, Empangeni, South Africa; 2 HIV Pathogenesis Programme, Doris Duke Medical Research Institute, Nelson R. Mandela School of Medicine, University of KwaZulu-Natal, Durban, South Africa; 3 Department of Paediatrics, University of KwaZulu-Natal, Durban, South Africa; 4 Department of Paediatrics, University of Oxford, Oxford, UK; 5 Africa Health Research Institute, Durban, South Africa; 6 Harry Gwala Regional Hospital, Pietermaritzburg, South Africa; 7 General Justice Gizenga Mpanza Regional Hospital, Stanger, South Africa; 8 Mahatma Gandhi Memorial Hospital, Durban, South Africa; 9 Clinical Immunology and Vaccinology Unit, IRCCS, Ospedale Pediatrico Bambino Gesù, Rome, Italy; 10 Probiomics S.r.l., Rome, Italy; 11 University of Rome Tor Vergata, Rome, Italy; 12 Fundación de Investigación Biomédica Hospital 12 de Octubre, Instituto de Investigación 12 de Octubre (imas12), Madrid, Spain; 13 Department of Pediatrics, Infanta Sofia University Hospital and Henares University Hospital Foundation for Biomedical Research and Innovation, Madrid, Spain; 14 Universidad Europea de Madrid, Madrid, Spain; 15 LMU University Hospital, Munich, Germany; 16 IrsiCaixa AIDS Research Institute, Barcelona, Spain; 17 University of Alabama at Birmingham, Birmingham, AL USA; 18 TU Dortmund University, Dortmund, Germany; 19 University of Padova, Padua, Italy; 20 Ragon Institute of MGH, MIT and Harvard, Boston, MA USA; 21 Nuffield Department of Medicine, University of Oxford, Oxford, UK; 22 Consorcio Centro de Investigación Biomédica en Red de Enfermedades Infecciosas (CIBERINFEC), Instituto de Salud Carlos III, Madrid, Spain; 23 Division of Infection and Immunity, University College London, London, UK; 24 University of California San Diego, San Diego, CA USA; 25 Catalan Institution for Research and Advanced Studies (ICREA), Barcelona, Spain; 26 Infectious Diseases and Immunity Department, University of Vic-Central University of Catalonia, Vic, Spain; 27 Birmingham Veterans Affairs Medical Center, Research Service, Birmingham, AL USA

**Keywords:** Predictive markers, HIV infections, HIV infections, Outcomes research

Correction to: *Nature Medicine* 10.1038/s41591-024-03105-4, published online 6 June 2024.

In the version of the article initially published, two of Javier Martinez-Picado’s affiliations (Catalan Institution for Research and Advanced Studies (ICREA), Barcelona, Spain and Infectious Diseases and Immunity Department, University of Vic-Central University of Catalonia, Vic, Spain) were missing and have now been added to the HTML and PDF versions of the article.

