# Supplementary material for: Sustained aviremia despite anti-retroviral therapy non-adherence in male children after in utero HIV transmission
Source: Nat Med. 2024 Jun 6;30(10):2796–804. doi: 10.1038/s41591-024-03105-4 (PMC11485204; doi:10.1038/s41591-024-03105-4)
Supplement: Supplementary file 1 — Supplementary Figs. 1 and 2 and Table 1. [file 41591_2024_3105_MOESM1_ESM.pdf]

nature medicine

Supplementary information https://doi.org/10.1038/s41591-024-03105-4

Sustained aviremia despite anti-retroviral therapy non-adherence in male children after in utero HIV transmission

In the format provided by the authors and unedited

Suppl Fig 1

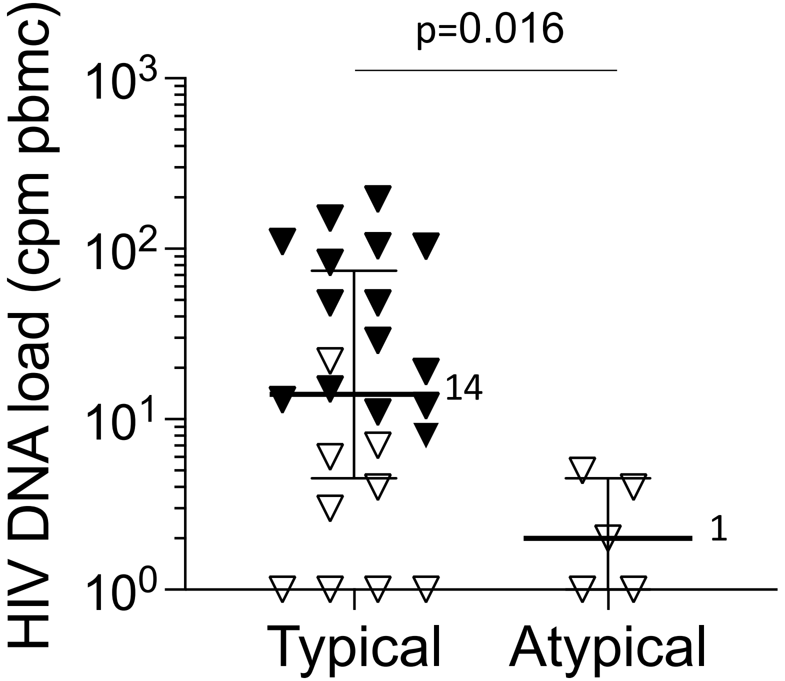

Suppl Fig 1. Total HIV DNA loads in 25 age-matched 'typical' children compared to the 5 'atypical' children analysed by ddPCR. The age of the children at which total DNA load was analysed was 46m median (IQR 37-48m) in the 'typical' group (n=25) and 41m (median; IQR 36-44m) in the 'atypical' group (n=5). Open symbols represent DNA loads that were undetectable or not significantly different from undetectable, solid symbols represent total HIV DNA detected (HIV DNA copies/million PBMC). P value determined using the two-sided Mann-Whitney U test.

Suppl Fig 2

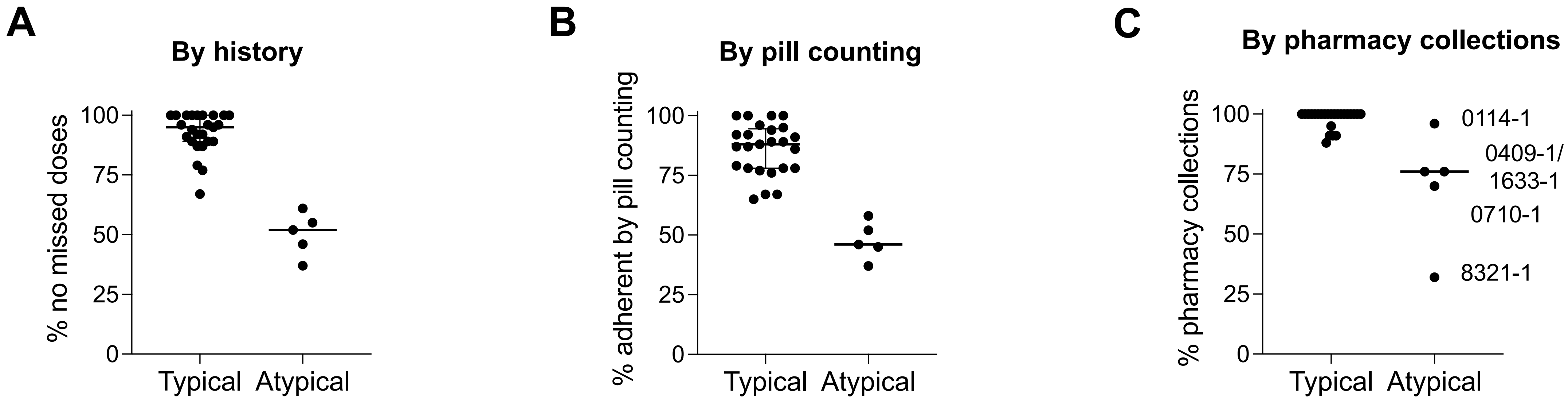

Suppl Fig 2. cART adherence estimates by history, pill counting and pharmacy collections. A. Percentage of timepoints at which no doses were missed according to history from the caregiver. **B.** Percentage of timepoints at which pill counting (volume of antiretroviral therapy) remaining corresponded to 100% cART adherence. In A-B, a missed clinic visit was counted as a timepoint when <100% cART adherence was achieved. **C.** Percentage of timepoints at which antiretroviral therapy was collected from the hospital pharmacy. In all panels, ‘Typical’ cases n=25, ‘Atypical’ cases n=5. Data for ‘Typical’ cases in panels A-B are presented as median and interquartile range.

Suppl Table 1

	**β coefficient**	**p value**
**Reference***	5.06	n/a
**IFN-α IC50**	+0.78	0.014
**Reference***	4.65	n/a
**IFN-β IC50**	+0.76	0.003

*Reference:
pVL when maternal $\log_{10}$ IC50 = 0

Suppl Table 1. Univariate linear regression analyses performed to determine the impact of IFNa IC50 and IFNb IC50 on MoM pVL. Univariate linear regression analyses were performed with *$\log_{10}$ (MoF pVL)* as response and either *$\log_{10}$ (IFNa IC50)* or *$\log_{10}$ (IFNb IC50)* as covariate, respectively. The p values in this regression model were determined using two-sided Student *t*-tests.
