# Supplementary material for: Sustained aviremia despite anti-retroviral therapy non-adherence in male children after in utero HIV transmission
Source: Nat Med. 2024 Jun 6;30(10):2796–804. doi: 10.1038/s41591-024-03105-4 (PMC11485204; doi:10.1038/s41591-024-03105-4)
Supplement: Supplementary file 2 — Reporting Summary [file 41591_2024_3105_MOESM2_ESM.pdf]

nature portfolio

Corresponding author(s): PROFESSOR PHILIP GOULDER

Last updated by author(s): May 22, 2024

Reporting Summary

Nature Portfolio wishes to improve the reproducibility of the work that we publish. This form provides structure for consistency and transparency in reporting. For further information on Nature Portfolio policies, see our Editorial Policies and the Editorial Policy Checklist.

Statistics

For all statistical analyses, confirm that the following items are present in the figure legend, table legend, main text, or Methods section.

n/a	Confirmed	
☐	☒	The exact sample size (*n*) for each experimental group/condition, given as a discrete number and unit of measurement
☐	☒	A statement on whether measurements were taken from distinct samples or whether the same sample was measured repeatedly
☐	☒	The statistical test(s) used AND whether they are one- or two-sided<br>*Only common tests should be described solely by name; describe more complex techniques in the Methods section.*
☐	☒	A description of all covariates tested
☐	☒	A description of any assumptions or corrections, such as tests of normality and adjustment for multiple comparisons
☐	☒	A full description of the statistical parameters including central tendency (e.g. means) or other basic estimates (e.g. regression coefficient) AND variation (e.g. standard deviation) or associated estimates of uncertainty (e.g. confidence intervals)
☒	☐	For null hypothesis testing, the test statistic (e.g. *F*, *t*, *r*) with confidence intervals, effect sizes, degrees of freedom and *P* value noted<br>*Give P values as exact values whenever suitable.*
☒	☐	For Bayesian analysis, information on the choice of priors and Markov chain Monte Carlo settings
☒	☐	For hierarchical and complex designs, identification of the appropriate level for tests and full reporting of outcomes
☐	☒	Estimates of effect sizes (e.g. Cohen's *d*, Pearson's *r*), indicating how they were calculated

Our web collection on statistics for biologists contains articles on many of the points above.

Software and code

Policy information about availability of computer code

Data collection	Excel for Mac v16.3, DataFax v 2016.0.0 and DF Discover version 5.1.0 were used to collect data from the Ucwaningo Lwabantwana study
Data analysis	GraphPad Prism version 7.0b R code (89. R Core Team R: A Language and Environment for Statistical Computing. R Foundation for Statistical Computing, Vienna, Austria. URL: https://www.R-project.org/. (2023). and FlowJo version 10.8.2

For manuscripts utilizing custom algorithms or software that are central to the research but not yet described in published literature, software must be made available to editors and reviewers. We strongly encourage code deposition in a community repository (e.g. GitHub). See the Nature Portfolio guidelines for submitting code & software for further information.

Data

Policy information about availability of data

All manuscripts must include a data availability statement. This statement should provide the following information, where applicable:

- Accession codes, unique identifiers, or web links for publicly available datasets
- A description of any restrictions on data availability
- For clinical datasets or third party data, please ensure that the statement adheres to our policy

There are no restrictions on the availability of materials or information relating to this manuscript. The data that support the findings in this study are available from the corresponding authors upon reasonable request.

Human research participants

Policy information about studies involving human research participants and Sex and Gender in Research.

Reporting on sex and gender	169 of the infants enrolled were females and 115 were males.
Population characteristics	The Ucwaningo Lwabantwana is a cohort of 284 in utero-infected children enrolled and followed in KwaZulu-Natal, South Africa from 2015-2023. At enrolment, the median age of the infants was 8d, and the median absolute CD4 count, CD4% and plasma viral load were 1987 copies/ul, 43.5% and 4250c/ml, respectively. At enrolment, the median age of the mothers was 25yrs, and the median absolute CD4 count, CD4% and plasma viral load were 475 copies/ul, 24% and 3400c/ml, respectively. The median age of the children enrolled at date 07/31/2023 was 4yrs 10m.
Recruitment	This has been described in detail in the text in the section entitled "Study subjects". All babies born to HIV positive mothers were tested at birth and if HIV positive were recruited for study. There was no selection bias in the recruitment process.
Ethics oversight	The Biomedical Ethics Research Committee, University of KwaZulu-Natal and the Oxford Research Ethics Committee approved the studies.

Note that full information on the approval of the study protocol must also be provided in the manuscript.

Field-specific reporting

Please select the one below that is the best fit for your research. If you are not sure, read the appropriate sections before making your selection.

☒ Life sciences ☐ Behavioural & social sciences ☐ Ecological, evolutionary & environmental sciences

For a reference copy of the document with all sections, see nature.com/documents/nr-reporting-summary-flat.pdf

Life sciences study design

All studies must disclose on these points even when the disclosure is negative.

Sample size	Sample sizes were determined by considerations of sample availability, study resources, and previous paediatric cohort studies we have undertaken in South Africa (eg Muenchhoff et al Sci Transl Med 2016) where the numbers studied had allowed statistically significant immune predictors of disease outcomes to be made. No statistical methods were used to predetermine sample sizes.
Data exclusions	No data were excluded from analyses
Replication	Determination of viral replication capacities, interferon-resistance IC50, ddPCR determinations of HIV DNA load are all highly reproducible and are assays with a high degree of precision. In the case of the VRC and ddPCR assays these are performed in triplicate. All attempts at replication were successful. In each case the reproducibility of the data from these assays has been demonstrated by repeat testing on a subset of the samples.
Randomization	All mothers and infants were from the same ethnic population (South Africa).
Blinding	All the analyses were done blinded to sex of the subject.

Reporting for specific materials, systems and methods

We require information from authors about some types of materials, experimental systems and methods used in many studies. Here, indicate whether each material, system or method listed is relevant to your study. If you are not sure if a list item applies to your research, read the appropriate section before selecting a response.

Materials & experimental systems

n/a	Involved in the study
☒	☐ Antibodies
☐	☒ Eukaryotic cell lines
☒	☐ Palaeontology and archaeology
☒	☐ Animals and other organisms
☒	☐ Clinical data
☒	☐ Dual use research of concern

Methods

n/a	Involved in the study
☒	☐ ChIP-seq
☐	☒ Flow cytometry
☒	☐ MRI-based neuroimaging

Eukaryotic cell lines

Policy information about cell lines and Sex and Gender in Research

Cell line source(s)	CEM-GXR cells were provided by collaborators Mark Brockman at Simon Fraser University, Canada. U87 MG/D1406 cell line were provided by collaborators John Kappes at University of Alabama at Birmingham, Birmingham, Alabama, USA. These cell lines are not commercially available.
Authentication	CD4 and CXCR4 expression on CEM-GXR and U87 cells were verified in-house by flow cytometry; and NL4-3 used as a positive control to assess cell permissiveness to virus infection.
Mycoplasma contamination	These cell lines were received mycoplasma, bacteria and fungi free; a new vial of cells was received and used. No in-house testing for mycoplasma contamination was done in addition.
Commonly misidentified lines (See ICLAC register)	No commonly misidentified cell line was used in this study.

Flow Cytometry

Plots

Confirm that:

☐ The axis labels state the marker and fluorochrome used (e.g. CD4-FITC).

☐ The axis scales are clearly visible. Include numbers along axes only for bottom left plot of group (a 'group' is an analysis of identical markers).

☐ All plots are contour plots with outliers or pseudocolor plots.

☐ A numerical value for number of cells or percentage (with statistics) is provided.

Methodology

Sample preparation	CEM-GXR cells were harvested daily, fixed in 2% PFA and acquired on the flow cytometer for quantification of GFP expression.
Instrument	BD LSR II
Software	FlowJo Software (Tree Star Inc., Ashland, OR).
Cell population abundance	GFP expression was quantified using CEM-GXR cell lines, no post-sorting of cells was involved.
Gating strategy	Live cells were identified based on SSC-A FSC-A distribution. FITC-A channel was utilized to quantify GFP expression. GFP+ populations were determined using an uninfected population of CEM-GXR as a negative control. No gating strategy was adopted here as a single population of GXR cells was used.

☐ Tick this box to confirm that a figure exemplifying the gating strategy is provided in the Supplementary Information.
